# Pancreatic Cancer and Gut Microbiome-Related Aspects: A Comprehensive Review and Dietary Recommendations

**DOI:** 10.3390/nu13124425

**Published:** 2021-12-10

**Authors:** Bartosz Kamil Sobocki, Karolina Kaźmierczak-Siedlecka, Marcin Folwarski, Viktoria Hawryłkowicz, Wojciech Makarewicz, Ewa Stachowska

**Affiliations:** 1 Scientific Circle of Oncology and Radiotherapy, Medical University of Gdansk, 80-210 Gdańsk, Poland; 2 Department of Surgical Oncology, Medical University of Gdansk, 80-210 Gdańsk, Poland; wojmakar@wp.pl; 3 Department of Clinical Nutrition and Dietetics, Medical University of Gdansk, 80-210 Gdańsk, Poland; marcinfol@gumed.edu.pl; 4 Department of Human Nutrition and Metabolomics, Pomeranian Medical University in Szczecin, 70-204 Szczecin, Poland; vik.hawrylkowicz@gmail.com (V.H.); ewast@pum.edu.pl (E.S.)

**Keywords:** pancreatic cancer, gut microbiota, prebiotics, probiotics, next-generation probiotics, synbiotics, postbiotics, fecal microbiota transplantation

## Abstract

Gut microbiota plays a significant role in the human body providing many beneficial effects on the host. However, its dysbiotic alterations may affect the tumorigenic pathway and then trigger the development of pancreatic cancer. This dysbiosis can also modulate the aggressiveness of the tumor, influencing the microenvironment. Because pancreatic cancer is still one of the most lethal cancers worldwide with surgery as the only method that influences prognosis and has curative potential, there is a need to search for other strategies which will enhance the efficiency of standard therapy and improve patients’ quality of life. The administration of prebiotics, probiotics, next-generation probiotics (*Faecalibacterium prausnitzii*, *Akkermansia muciniphila*), synbiotics, postbiotics, and fecal microbiota transplantation through multiple mechanisms affects the composition of the gut microbiota and may restore its balance. Despite limited data, some studies indicate that the aforementioned methods may allow to achieve better effect of pancreatic cancer treatment and improve therapeutic strategies for pancreatic cancer patients.

## 1. Introduction

According to the American Cancer Society report from 2019, pancreatic cancer is one of the most common cancers [1,2]. Two main types of pancreatic cancer are distinguished, such as pancreatic ductal adenocarcinoma—PDAC and pancreatic neuroendocrine tumor affecting less than 5% of patients. These tumors are associated with different biology, aggressiveness, and outcome [3]. In our review, we would like to focus on PDAC. It is estimated that over 75% of patients are in the advanced stage at the time of PDAC diagnosis [4]. The development of pancreatic cancer is triggered through many factors including genetic background (mutations of genes), lifestyle (high-fat diet, smoking), gut microbiota dysbiosis, the occurrence of other diseases/conditions (obesity, type 2 diabetes, pancreatitis), and many others [2,5,6,7,8].

Due to the poor long-term outcomes of PDAC treatment and lack of systemic therapy efficiency, there is a need to find new therapeutic strategies and supportive therapies to improve patients’ quality of life. Recently, growing attention towards the association between pancreatic cancer and gut microbiota-related aspects has been observed. In pancreatic cancer multiple bacterial, fungal, and viral gut microbiota imbalance is observed [9]. Therefore, the modulation of gut microbiota and restoration of its diversity and balance may be crucial.

In the present review, we focused on the role of the gut microbiome in pancreatic carcinogenesis. We discussed not only bacterial gut microbiota alterations but also the role of its viral and fungal parts. Additionally, from an interdisciplinary point of view, we presented available therapeutic methods, which modify gut microbiota in pancreatic cancer through administration of prebiotics, probiotics, synbiotics, postbiotics, next-generation probiotics, and fecal microbiota transplantation (FMT). We tried to summarize possible implementations of the mentioned methods in clinical practice. Finally, we also presented ongoing trials, registered in ClinicalTrials.gov.

## 2. The Link between Gut Microbiota and Pancreatic Carcinogenesis. Intra-Tumour Microenvironment

### 2.1. Bacteria

The association between bacteria and pancreatic cancer has been analyzed many times, in comparison to fungi and viruses. The importance of the microbiome in the pathogenesis of PDAC was emphasized by many studies reporting that activation of the pattern recognition receptors (PRRs) (which are responsible for the transmission of inflammation) by microbial pathogens stimulates tumorigenesis. By analogy, the deficiency of some PRRs pathways such as Mincle and Toll-like receptors 4,7,9 were associated with inhibition of PDAC progression in mice models [10,11,12,13,14,15].

Pushalkar et al. conducted a comprehensive study that concluded that gut and intra-tumor microbiome have to be taken into consideration together [15]. In the mice model, fluorescently-labeled *Enterococcus faecalis* and *Escherichia coli* introduced via oral cavity migrated into the pancreas and had a direct impact on the microenvironment of this organ [15]. Moreover, in the group of antibiotic-treated mice, it was reported that the gut microbiome in Pdx1Cre; LSL-KRASG12D; p53R172H mice were more able to migrate than in wild-type mice [15]. Gut microbiota can migrate into pancreatic tissue via portal circulation or mesenteric lymph nodes [16,17]. It is noteworthy that the composition of gut microbiota is closely related to gut permeability [17]. Concluding, we should not treat the microbiome like a static population closed in different, separate compartments, but rather like dynamic and able to migrate population interacting with tumor microenvironment and microbiome in other locations. Although currently, we know that microbiome changes in the pancreatic microenvironment and its ability to migrate might be a key. Some new, detailed, and mechanism-focused studies investigating how the microbiome affects PDAC after migration should be done.

In addition, the study of Pushalkar et al. showed in both humans and mice an increased bacterial abundance (bacterial DNA content) in PDAC tissue in comparison to the healthy pancreas tissue (confirmed by 16S rRNA FISH and qPCR) were observed [15]. Moreover, it revealed that the PDAC microenvironment consists of 13 distinct phyla with the domination of *Proteobacteria* (45%), *Bacteroides* (31%), and *Firmicutes* (22%) which are relevantly different than in normal pancreas. The gut microbiome was dominated by Firmicutes and Bacteroides in control as well as in PDAC patient-derived fecal samples. However, there was a difference in the abundance of Proteobacteria, Synergistetes, and Euryarcheota [15]. The impact of bacteria on the progression of pancreatic cancer was observed both in pre-invasive and invasive models whereas oral administration of antibiotic reduced tumor size by approximately 50% in mice model with a similar effect of antibiotic application in KRAS wild-type Pan02 cells. Consequently, this study proved the association between the microbiome and induction of peritumoral immune suppression [15]. In p48Cre; LSL-KrasG12D (KC) pancreas model microbial ablation caused the increase in a number of intra-tumoral T cells, reduction of the population of myeloid-derived suppressor cells (MDSC), promotion of the domination of pro-inflammatory M1-like tumor-associated macrophages over M2-anti-inflammatory subtype, increased CD8:CD4 ratio and higher levels of pro-inflammatory cytokines, such as TNF-α or IFNγ [15]. A similar impact on the orthotopic Pan02 model was observed [15]. It is worth pointing out that macrophages treated with an extract from gut bacteria (from hosts with PDAC) had significantly reduced potential for activation of T cells. In addition, bacterial ablation was associated with PD-1 up-regulation [15]. This result may be promising and valuable regarding PD-1-associated therapies. Another human study indicated that *Proteobacteria* and *Firmicutes* dominance in gut microbiota was observed in early PDAC. Moreover, significantly elevated levels of substances in polyamine and nucleotide biosynthetic pathways were reported. These metabolic products are utilized during intensive divisions of cancer cells and their levels are strongly correlated with microbial composition changes [3]. A study by Farell et al. estimated that a combination of two oral and bacterial biomarkers (*Neisseria elongata* and *Streptococcus mitis*) differentiated patients with PDAC from healthy subjects with a 96.4% sensitivity and 82.1% specificity (area under the curve value of 0.9) whereas the study of Castillo et al. reported that *Fusobacterium* abundance was significantly higher in the intra-tumor microenvironment of PDAC patients in comparison to control, contrary to decreased *Lactobacillus* [18,19]. Another study by Matsukawa et al. gave an insight into microbiota composition differences between PDAC and healthy subjects and showed that the following species were significantly more abundant in PDAC than in control: *Klebsiella pneumoniae*, *Clostridium bolteae*, *C. symbiosum*, *Streptococcus mutans*, *Alistipes shahii*, 4 *Bacteroides* species, 2 *Parabacteroides* species, and 2 *Lactobacillus* species whereas the fecal abundance of *Bifidobacterium animalis*, *Collinsella aerofaciens*, *Eubacterium ventriosum*, *Klebsiella pneumoniae*, *Roseburia intestinalis*, and *Streptococcus thermophilus* can be treated as prognostic factors for PDAC [20]. Last, but not least, a study indicated a significant reduction of the Firmicutes phylum and an increase of Proteobacteria only among PDAC patients. In addition, *Eubacterium rectale*, *Eubacterium ventrisum*, and *Odoribacter splanchicus* were the most relevant biomarkers in the differentiation of PDAC patients from healthy subjects and patients suffering from autoimmune pancreatitis. Moreover, functional analysis showed an increase of molecules associated with bacterial virulence and a decrease of short-chain fatty-associated molecules. Gas chromatography revealed a relevant decrease in butyrate fecal concentration [21]. Knowing that microbial composition is different in early and advanced PDAC as well as in the comparison between PDAC and control, microbial dysbiosis can be treated as a potential diagnostic marker and prognostic factor for this cancer. Taking this into consideration, new clinically orientated human studies targeting interactions of the immune system with gut and intra-tumor microbiome are needed. New meta-analyses validating and summarizing studies would be also valuable.

One of the human studies using 16S rRNA gene amplification and sequencing methods was conducted and showed that presence in oral cavity phyla such as *Porphyromonas gingivalis* and *Aggregatibacter actinomycetemcomitans* were associated with a higher risk of PDAC whereas phylum like *Fusobacteria* decreased this risk [16]. Another study using human oral microbe identification microarrays pointed out that *Neisseria elongata* and *Streptococcus mitis* species had significantly decreased levels in PDAC in comparison to control [18]. Studies like that emphasize the role of periodontal diseases treatment in PDAC and other cancers prevention.

Another conclusion can be drawn from the study which compared two cohorts of patients: long-term survivors (who survived more than 5 years after surgery, LTS) and short-term survivors (who survived less than 5 years after surgery, STS) using 16S rRNA gene sequencing. This study revealed that a number of species were significantly higher in LTS than in the STS cohort and that patients with higher diversity of microbiome had relevantly prolonged overall survival [22]. Microbiome compositions in LTS and STS groups were different. The LTS cohort was dominated by *Alphaproteobacteria*, *Sphingobacteria*, and *Flavobacteria* at the class level whereas the STS cohort was by Clostridia and Bacteroidea. In addition, in the LTS patients, higher abundance of *Proteobacteria*, *Actinobacteria*, and one species *Bacillus clausii* were observed whereas there was a lack of a similar effect in the STS group. Moreover, this higher abundance was also significantly associated with a better prognosis [22]. This study confirmed the association between microbiome and immune system reported by Pushalkar et al. The greater densities of CD3+, CD8+ T cells, and Granzyme B+ were observed in the LTS cohort compared to the STS cohort. There was also consistency between these studies in lack of significant differences in levels and activity of regulatory T cells. However, when Pushalkar et al. reported the significant role of macrophages and reduction of the MDSC population, there was a lack of significance in the case of Riquelme et al. study [15,22]. In addition, this study revealed that in the cohort of LTS patients, xenobiotics biodegradation and lipid metabolism pathways were stimulated whereas in STS cohort similar effect was observed for synthesis and processing of proteins and genetic information, energetic and nucleotide metabolism, replication, and repair. Metabolic pathways active in the LTS group were associated with better outcomes [22]. Another study indicated that gut microbiome depletion caused a significant reduction of the tumor burden in the pancreatic cancer mice model and was associated with a decrease in liver metastases in the PDAC model. However, a similar effect was not observed in Rag1 knockout mice (with a lack of mature T and B lymphocytes). It emphasizes the necessity of active participation of adaptive immunity and that the effect of tumor reduction was not caused by the direct cytotoxic effect of antibiotics on tumor cells [23]. Essential conclusions can be also drawn from bioinformatic analysis conducted by Luo et al. This study based on the Gene Expression Omnibus database investigated 3 differentially expressed genes in PDAC (TUBB, TUBA4A, TLR5) and showed that these genes were mainly enriched in the case of pathogenic *Escherichia coli* infection. In addition, survival analysis showed that one of these genes, TUBB (tubulin, class I) may be involved in carcinogenesis and progression, activating TUBB/Rho/ROCK signaling pathway and inhibiting immune response [24]. This molecular pathway should be evaluated as a potential target therapy for PDAC in future studies. Interestingly, the study which used contaminated (stended), sterile (nonstended), and sterile (but preincubated) bile samples collected during pancreaticoduodenectomy revealed that all bile samples relevantly reduced peritoneal metastasis of Panc02 cells in mice whereas all sterile bile samples reduced in vitro survival of pancreatic cancer cell lines (AsPC1, CFPAC, Panc1). In addition, preincubation of sterile bile with *Streptococcus oralis* and *Enterococcus faecalis* resulted in the modified antitumour activity of sterile bile which indicated the impact of gut bacteria on antitumour components presence in bile [25] Another study investigating the Italian cohort reported that an increase of *E. coli* in bile resulted in shortened survival [26].

**Figure 1:**
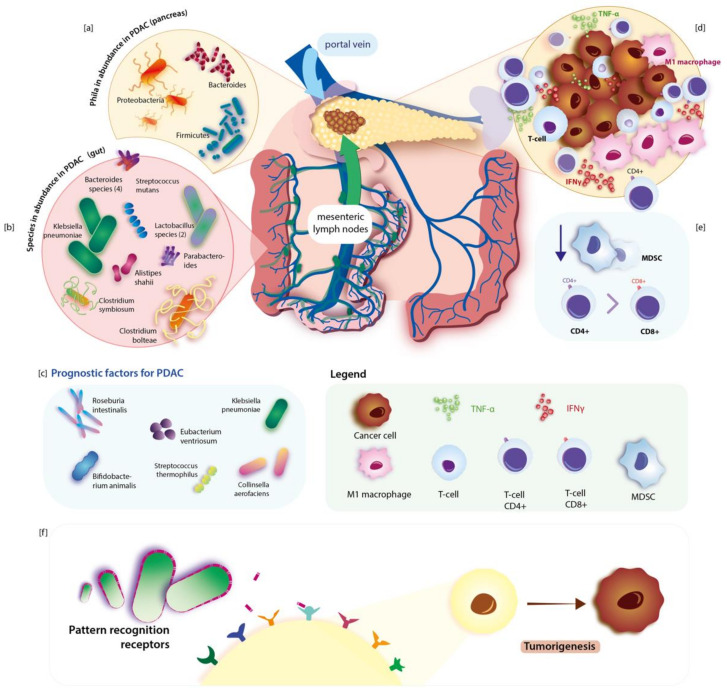
Dysbiotic alterations of the gut microbiome in pancreatic cancer regarding immunological response. Species of bacteria that are abundant in the gut in PDAC are listed in section (**b**). Some of them can be used as prognostic factors of PDAC and are found in section (**c**). Gut microbiota can migrate into the pancreas via a portal vein or mesenteric lymph flow. Phyla that are abundant in the pancreas during PDAC are illustrated in section (**a**). Sections (**d**) and (**e**) illustrate peri-tumoral immunological response in PDAC: the increase of intra-tumoral T-cells, pro-inflammatory M1-like tutor associated macrophages, CD4, levels of pro-inflammatory cytokines (such as TNF-α, IFNγ) and reduction of MDCS. Pathogens can stimulate tumorigenesis by activating inflammation-inducing PRRs. The mechanism of pathogenesis of PDAC is illustrated in section (**f**). Own elaboration based on literature.

Another interesting context is the association of gut and intra-tumor bacteria with treatment outcomes and its predictive role. The study conducted by Geller et al. indicated that the *Gammaproteobacteria* class has the ability to metabolize gemcitabine (a chemotherapeutic agent which is used in the treatment of advanced PDAC) and that presence of *Gammaproteobacteria* in tissue specimens was associated with resistance to this drug [27]. On the other hand, gemcitabine can considerably change microbiota composition in the gut and subsequently cause an increase in bacterial species associated with inflammation [28]. Another basic combination is known as FOLFIRINOX, which is used in advanced PDAC, and may also be affected by microbiota. Ida et al. showed that efficiency of two drugs from this combination (oxaliplatin and cisplatin) in MC38 and B16 tumor-bearing mice was enhanced by a healthy microbiota (through stimulation of the reactive oxygen species released from myeloid cells) whereas a decrease of efficiency was correlated with reduced and eliminated microbiota [29]. Investigation and deep understanding of interactions of microbiome with systemic therapy drugs may help in improving the outcome of patients in the future. Studies like AGITG MASTERPLAN may give an insight into these specific and complex mechanisms and provide new targets for effective treatment in such devastating diseases as PDAC [30]. Interestingly, Dong et al. reported that also metformin may have a chemopreventive effect and eliminate PDAC formation in KC mice through changes in duodenal microbiome composition [31]. In addition, a study by Kesh et al. (type 2 diabetes—T2D mouse model) showed that T2D induced microbiome dysbiosis caused by hyperglycemia may result in impaired response to gemcitabine/paclitaxel combination [32]. In the Maletzki et al. experiment authors infected Panc02 tumor with Streptococcus pyogenes. Treatment with that pathogen resulted in completed tumor regression with parallel massive leucocyte infiltration into the tumor and elevation of pro-inflammatory cytokines in a syngeneic mouse model [33]. Figure 1 presents bacterial gut microbiome changes and their link to PDAC.

### 2.2. Fungi

The link between pancreatic carcinogenesis and fungal microbiota imbalance is still not described well. The gastrointestinal tract is dominated by bacteria and most studies have focused on the bacterial part of gut microbiota. Bacteria constitute around 98% of microbiota whereas only 0.2% of microorganisms in the gut are fungi [6]. *Candida*, *Saccharomyces*, and *Cladosporium* are the most common fungal genera which reside in the healthy human gut [8]. The composition of fungal gut microbiota depends on dietary factors (mainly consumption of carbohydrates) [8]. It plays multiple functions in the human body regarding maintenance of gut homeostasis, production of metabolites, affecting gut immunity, interaction with microbes, and many others [2]. Dysbiotic changes of fungal gut microbiota affect pancreatic carcinogenesis [2]. Recently, in 2019 Aykut et al. have shown that it promotes pancreatic carcinogenesis through activation of mannose-binding lectin (MBL) [9]. Notably, fungi can migrate from the gut lumen into the pancreas causing alterations of the pancreas environment [9]. Fungi and other microbes may affect the host’s immune system [34]. Fungi may accelerate pancreatic cancer and they may be involved in carcinogenesis via several species-dependent mechanisms [2,35]. For instance, *Malassezia* stimulates the production of pro-inflammatory cytokines and activates mast cells [36,37]. Additionally, it accelerates tumorigenesis through activating the C3 complement-mannose-binding lectin pathway [34]. Another fungal genus—*Candida* triggers inflammation and increases the proliferation of myeloid-derived suppressor cells [2].

### 2.3. Viruses

Associations between viruses and PDAC are not sufficiently proven. There is still a lack of studies comprehensively describing this axis and evaluating large cohorts of subjects. However, studies indicated that some viruses may be important as risk factors and prognostic factors. For instance, a meta-analysis conducted by Arafa et al. confirmed that hepatitis C virus infection increases the risk of pancreatic cancer like in hepatocellular carcinoma [38]. Similarly, the investigation of Li et al. confirmed the role of hepatitis B virus infection in the increase of PDAC risk [39]. Although another meta-analysis of Liu et al. reported similar results, it also reported significant heterogeneity in studies. In addition, when authors provided detailed analysis according to regions, it was revealed that significant impact of this virus was reported only in Asia and Oceania when in Europe it was non-relevant [40]. Taking heterogeneity into consideration, new data should be provided in that area to give a clear answer about that virus. In the case of mechanism, Jin et al. suggested that in the case of PDAC pathogenesis the link between pancreatitis and hepatitis B virus (HBV) may be essential [41]. Moreover, Chen et al. using cell lines reported that HBV virus and HBx expression may induce cell proliferation, migration, and epithelial-mesenchymal phenotype in pancreatic cancer. Significant positive correlations between HBx expression and typical cancer-associated molecules like ErbB4, TGF-α levels were observed. In addition, HBx promoted the increase in phosphorylation of AKT, a molecule important in the activation of cell proliferation and migration [42]. Walter et al. provided an insight into the Newcastle disease virus and showed using that normal human cell lines (keratinocytes, fibroblast, pancreatic ductal cells, vascular endothelial cells) and 7 different pancreatic tumor cell lines that the pancreatic tumor cells were killed more than 700 times often than control cells [43].

## 3. Therapeutic Modulation of Gut Microbiota in Pancreatic Cancer

### 3.1. Prebiotics

Prebiotics can be defined as nutrients that are degraded by gut microbiota and may affect not only the intestinal microenvironment but also distant organs [44]. It may be one of the reasons why research interests in this topic are developing, especially in terms of cancer. In PDAC there is still a small number of studies that provide interesting conclusions. Trivieri et al. used xenograft mice model confronted with pancreatic cancer gene expression dataset (GSE16515) and investigated the impact of high levels of prebiotic resistant starch diet (RSD) on miRNA expression profiles in tumor tissues. Interestingly, a diet rich in those substances was associated with dysregulation of 19 miRNAs genes expression in comparison to control. To determine the biological functions of the differentially expressed miRNA genes (in comparison between mice fed with RSD and control) authors conducted predictive analysis by ingenuity pathways analysis. It was revealed that part of genes participating in the regulation of processes such as the development of carcinoma, inflammatory response, abdominal cancer, metabolic disease, growth, invasion, and metastasis were downregulated in a group of mice fed with RSD in comparison to control. In addition, genes participating in the synthesis of carbohydrates, glucose metabolism disorder, and cell death of cancer cell lines were significantly upregulated in mice fed with RSD. Besides, IPA network analysis of PDAC signaling showed up-regulation of TGFBR2, AKT, and in mice fed with RDS. To determine the association between 19 differentiated expressed miRNA and the prognosis of PDAC authors performed TCGA analysis. Results of the analysis revealed that four of miRNA up-regulated in mice fed with RSD such as miRNA-375, miRNA-148a-3p, miRNA-125a-5p, miRNA-200a-3p were significantly associated with PDAC prognosis. The authors based on TCGA proved that higher expression of those genes is associated with significantly better outcomes and prolonged overall survival, concluding beneficial value of RSD in PDAC [45]. However, we need to remember that this conclusion should be confirmed on numerous and homogenous groups of patients in order to avoid bias being a consequence of the indirect conclusion. Another study confirmed that the RSD diet can modulate gene expression and in addition, metabolomic profile in pancreatic cancer xenograft mice. Detailed analysis of RNA-Seq results showed dysregulation of 25 genes in mice fed with RSD in comparison to those with a control diet. Moreover, LC-MS analysis revealed dysregulation of six serum metabolites levels. Bioinformatics analysis predicted that functions of these genes were associated with insulin receptor signaling, circadian rhythm signaling, cancer drug resistance, cell death and survival, gene expression, and neurological diseases. In the group of metabolites acetylcarnitine, arginine, aspartic acid, hypoxanthine, inosine, and xanthine levels were significantly decreased whereas glutamine level was relevantly increased. There is a widely known fact that hypoxanthine, inosine, and xanthine are purines that play a role as a ‘fuel’ in increased cancer metabolism [46,47]. Panebianco et al. suggested that purines decrease in blood in mice fed with RSD may interfere with cancer cells proliferation. However, this study does not provide insight into purines directly in the tumor. In the case of glutamine, the authors suggested that an increase in blood may be associated with the lower glucose availability in mice fed with RSD and subsequent inhibition of glutamine uptake and utilization by the tumor [46]. In order to deeply understand and explain the potential mechanism and interactions of RSD with the tumor microenvironment and blood components, we need new studies. Clinically orientated studies showing the impact of RSD on metabolome and gene expression in PDAC and association with overall survival would be highly valuable, indicating potential targets for therapy.

### 3.2. Probiotics and Next-Generation Probiotics

The definition of probiotics is “live microorganisms which when administered in adequate amounts confer a health benefit on the host” [48]. They modify gut microbiota and may have an impact on pancreatic carcinogenesis as well as the efficiency of pancreatic cancer treatment. Notably, the effect of probiotics is strongly strain-dependent.

Pancreatoduodenectomy is associated with complications including infections, pancreatic fistula, delayed gastric emptying, and others [49]. In the Nomura et al. study, the impact of probiotics (*Enterococcus faecalis*, *Clostridium butyricum*, and *Bacillus mesentericus*) on infectious complications after pancreatoduodenectomy was assessed. This study enrolled 64 participants (probiotics *n* = 30, control subjects *n* = 34) [50]. The incidence of infectious complications was significantly lower in probiotics recipients in comparison to control group (23% vs. 53%, *p* = 0.02; respectively) [50].

It should be emphasized that the molecules which are derived from probiotics also play a significant role in acting against pancreatic cancer [20]. Ferrichrome is a molecule received from probiotics that suppressed the growth of refractory pancreatic cancer cells. Its mechanism is based on the inhibition of cancer cells progression and cell cycle dysregulation by activating p53 [51]. Ferrichrome is derived from probiotic strain *Lactobacillus casei* ATCC334 [52] and may act against not only pancreatic cancer, but also gastric as well as colon cancer [52,53].

Next-generation probiotics are identified using next-generation sequencing and bioinformatics tools [54]. *Faecalibacterium prausnitzii*, *Parabacteroides goldsteinii*, *Bacteroides fragilis*, *Akkermansia muciniphila*, *Prevotella copri*, *Christensenella minuta*, *Bacteroides thetaiotaomicron* are recognized as the next generation probiotic candidates [55]. Faecalibacterium prausnitzii is one of the most important butyrate-producing bacteria [56]. Recently, Zhou et al. have shown that patients with pancreatic ductal adenocarcinoma have a reduced level of *Faecalibacterium prausnitzii*, *Eubacterium rectale*, *Roseburia intestinalis,* and a significant increase of *Proteobacteria phylum* (mainly Gammaproteobacteria) [21]. Therefore, butyrate-producing bacteria are significantly decreased in these patients. *A. muciniphila* is a Gram-negative bacterium that plays a significant role in the human body, such as maintenance of intestinal immunity, regulation of cytokines release, acting against pathogens, and many others [57,58]. In Liu et al. study it was noted that live *A. muciniphila* inhibits the proliferative activity of INS-1 (rat pancreatic islet cell tumor cells) (*p* < 0.005) [59]. Next-generation probiotics are not well-studied, not only in the context of pancreatic cancer but overall. Nevertheless, they may open new perspectives for cancer patients.

### 3.3. Synbiotics

Synbiotics can be defined as products consisting of both probiotic and prebiotic. The reason for their use may be caused by a short survival of probiotics in the gastrointestinal tract [60]. Still, there is a lack of studies describing synbiotics in PDAC in the literature. We can try to draw a conclusion from studies concerning acute pancreatitis. A prospective, randomized, double-blind study compared patients receiving only prebiotics containing inulin, beta-glucan, resistant starch, and pectin vs patients with these prebiotics and in addition four different *lactobacilli* preparations with 10^10^ CFU. In the group receiving synbiotics compared to control significant results such as lower total incidence of systemic response syndrome, lower rate of late (over 48 h) organ failure, fewer patients recovering with complications, and following non-significant: lower incidence of multiorgan failure, septic complications, and mortality were observed [61]. Although chronic pancreatitis is associated with pancreatic cancer development and its progression as we reported above, any conclusions from that disease for PDAC may be associated with inestimable bias. We need new studies specifically in the context of PDAC.

### 3.4. Postbiotics

The aforementioned probiotics may play a significant role in supporting the treatment of pancreatic cancer. The benefits of probiotics in various medical conditions have been confirmed by several published meta-analyses [62,63]. Single publications indicate a potentially beneficial role of probiotic therapy in animals with pancreatic cancer, however, no meta-analysis confirms the benefit of probiotic therapy in patients with pancreatic cancer.

The results of single studies indicating risks in these patients call into question the safety of probiotics in high-risk patients (e.g., acute pancreatitis) [64], therefore, some researchers are considering replacing probiotics with the use of postbiotics in the treatment of these patients. However, postbiotics-related aspects are rapidly developing but it is still a very poorly understood area [65]. The exact definition of postbiotics is not yet established. Postbiotics can include bacterial metabolites beneficial to the host, such as short-chain fatty acids, exopolysaccharides, vitamins, phenols, bacterial lysates, supernatants, enzymes, and cell wall fragments [66]. It appears that bacterial metabolites can affect the intestinal microbiota of patients more safely than probiotics. Postbiotics may be such “beneficial” metabolites of the intestinal microbiota [67].

Although the number of studies regarding the role of postbiotics in pancreatic cancer patients is strongly limited, it is assumed that mechanisms of action of some postbiotics may be based on suppressing the inflammation, restoration of the gut barrier integrity, or the exertion of selective cytotoxicity against tumor cells [65]. An example of this first postbiotic effect may be the protein p40 secreted by *Lactobacillus rhamnosus* GG that inhibits epithelial gut barrier disruption (induced by inflammatory cytokines) [68,69]. Other researchers have demonstrated anti-inflammatory properties of supernatants obtained from cultures of *Bifidobacterium breve* CNCM I-4035 [70] or *other Lactobacillus casei*, *Lactobacillus reuteri*, *Lactobacillus acidophilus*, *Lactococcus lactis*, and *Saccharomyces boulardii* [71].

### 3.5. Fecal Microbiota Transplantation

Fecal microbiota transplantation (FMT) is a treatment strategy focused on gut microbiota modulation, with developing potential for clinical use in many diseases. The main aim of this procedure is the restoration of a more favorable microbial composition. This composition may be associated with modulation of natural anti-cancer response and outcome of immunotherapy [72,73,74]. In PDAC Riquelme et al. investigated the possibility of active modification of the tumor microbiome. Authors performed FMT from patients with advanced PDAC into mice treated with antibiotics before, transferring fecal material three times a week and repeating the sequence after 2 weeks. Then orthotopic implantation of syngeneic cancer lines from genetically engineered mice was performed. Interestingly, 5 weeks after transplantation about 40% of gut microbiota consisted of human donor origin species [22]. On the other hand, human-derived bacteria were only about 5% tumor microbiome while the remaining 20% consisted of the basal murine gut microbiome. The other 70% did not represent the gut microbiome. In addition, the authors revealed that the *Clostridiales* class presence was specifically and relevantly increased in the tumor microenvironment. A positive correlation was observed between the presence of this class in tumor and human origin samples [22]. After that, human samples from the group of patients such as advanced PDAC patients (APDAC), patients over 5 years after resection (5AP), and healthy controls (HC) were obtained. A similar procedure as above was repeated. After 5 weeks authors took samples and showed that gut microbiota beta-diversity distinguished three types of mice reflecting a group of patients. Moreover, a significantly greater reduction of tumor in mice who received FMT from the 5AP group was observed, comparing to HC and APDAC. In addition, the tumor was relevantly larger in mice that received FMT from HC than in the APDAC group. Besides, this study showed that short-term antibiotics administration in mice after FMT from the 5AP group resulted in a significantly larger tumor in comparison to lack of antibiotics treatment, which suggests that bacteria play a major role in that matter [22]. To conclude, bacterial composition associated with PDAC may promote tumor development, FMT from patients surviving long-term may be beneficial and should be investigated in future studies. Last, but not least, results concerning FMT in this study addressed tumor infiltrates. It was revealed with flow cytometry that tumors from mice that received FMT from the 5AP group were significantly more infiltrated by CD8+ T cells (as well as activated T cells CD8+ IGNγ+, T cells) whereas mice that received FMT from the APDAC group were more infiltrated by CD4+FOXP3+ and MDSC in comparison to other groups. In addition, mice receiving FMT from 5AP had higher levels of interferon-γ and interleukin-2 than those from the APDAC group. Depletion of CD8+ caused a reduction of these effects, indicating its major role [22]. Still, there is a lack of specific analysis of FMT along with the survival in PDAC. Currently, one early phase I trial related to FMT is registered in ClinicalTrials.gov (Identifier: NCT04975217).

### 3.6. Short Summary

The direct comparison of therapeutic strategies may be difficult. As we observed in the literature, when prebiotics, probiotics, and fecal microbiota transplantation are relatively well described, next-generation probiotics, synbiotics, and postbiotics effectiveness are less evidenced. Moreover, currently, there is a lack of clinical trials and other studies comparing the effectiveness of different methods in PDAC. To sum up the above-mentioned studies, prebiotics commonly affects different miRNA expressions and modulate levels of metabolites such as purines or amino acids. Describing probiotics, their effectiveness is essential in reduction of pancreatoduodenectomy complications. Molecules which are derived from probiotics may directly suppress the growth of pancreatic cancer cells. Next-generation probiotics open new perspectives for cancer patients. They have anti-proliferative properties as well as regulate intestinal immunity, cytokine release, and act against pathogens. In case of synbiotics, we have limited knowledge about their role in PDAC. What is known, is that they decrease the risk of severe complications in acute pancreatitis. Postbiotics seem to be less safe than probiotics. They probably suppress inflammation, participate in the restoration of the gut barrier integrity and have selective cytotoxicity against tumor cells. Lastly, fecal microbiota transplantation in PDAC patients caused reduction of tumor size (parallel antibiotic administration seems to be synergistic) and changed in a favorable way the immunological profile of tumor.

## 4. The Registration of Studies Regarding Pancreatic Cancer and Gut Microbiota-Related Aspects in the System

Nowadays, 10 studies (Table 1) regarding pancreatic cancer and gut microbiome-associated aspects are registered in the ClinicalTrials.gov system (accessed on 26 August 2021, terms: pancreatic cancer; gut microbiome). Most of them are observational studies.

## 5. Clinical Nutrition in Pancreatic Cancer

### 5.1. Nutritional Assessment and Support

The composition, as well as the activity of the gut microbiome, depends on many factors including the intake of antibiotics, surgical and pharmacological treatment (e.g., chemotherapy), age, and many others [75]. It is noteworthy that nutrition extremely affects the gut microbiome. An appropriate diet may positively stimulate the growth of beneficial microbes whereas the reduction of the number of pathogens maintains gut homeostasis [76]. Moreover, dietary factors stimulate the production of short-chain fatty acids. The nutritional guidelines for PDAC patients are presented below.

Multifactorial support for cancer patients is of great importance in improving quality of life (QoL) and prolonging survival, also in PDAC patients [77]. Early supportive care in patients with cancer is gaining more and more recognition in recent years due to its potential for improvement of patient outcomes, longer survival, and QoL [78,79]. It is said that the majority of patients will develop cachexia and suffer from involuntary loss of body weight, which decreases the survival of patients, treatment response, and their QoL [77,78,79,80,81]. It should be emphasized that sarcopenia and malnutrition are strongly associated with decreased chemotherapy tolerance, short survival, postsurgical complications, and poor QoL in patients with PDAC [77,82,83]. This indicates that maintaining a stable weight and muscle mass improves the prognosis [84].

Malnutrition is a frequently reported problem among pancreatic cancer (PC) patients. According to the literature, about 80% of PC patients experience loss of weight at the time of diagnosis and more than 30% of patients have lost >10% of their body weight [85]. Approximately two-thirds of PC patients manifest malnutrition and anorexia during their first medical consultation [86,87]. What is more, the majority of chemotherapy patients (about 70%) developed malnutrition [88].

Therefore, nutritional support in patients with pancreatic cancer is extremely important, also in the early stages of the disease. Clinical nutritionists and dietitians should be a part of a therapeutic team to provide patients with support and nutritional treatment [89,90]. The importance of preoperative dietetics consultation in cancer patients is high as lack of nutrition support before surgery is associated with increased preoperative weight loss, risk of malnutrition, and its postoperative implications [91]. The very first step of nutritional aid is a proper screening test, performed by specialists using validated tools at the time of diagnosis and regularly during therapy. The recommended screening tools are the Malnutrition Universal Screening Tool (MUST), the Nutritional Risk Screening 2002 (NRS 2002), the Malnutrition Screening Tool [MST], the Mini Nutritional Assessment [MNA]). Once the nutritional risk is confirmed, the nutritional treatment plan should be implemented as soon as possible by medical personnel specialized in clinical nutrition for cancer patients [89]. The applied nutritional intervention should be individually tailored to the patient, depending on the location of the tumor, nutritional status, clinical condition, and planned oncological treatment.

Adequate nutritional support is required for malnourished patients and at risk of nutrition, especially when oral energy intake is already insufficient or suspected to be insufficient (patient consumes <60% of estimated caloric intake for >7 days) [92,93]. Oncological malnourished patients waiting for surgery must receive pre-operative nutritional support for >7 days, even at the cost of delaying surgery, as this procedure improves postoperative outcomes [94].

The intake of oral nutritional supplements (ONS) may affect the gut microbiome. Currently, in ClinicalTrials.gov is the registered study (Identifier: NCT04980950) that assesses the role of immunonutrition (given orally as ONS or enterally) in modulating gut microbiome in the perioperative period. Notwithstanding, this study regards only patients with gastric as well as colorectal cancer. The use of ONS should be introduced both to treat malnutrition and the risk of its occurrence. In a study conducted to investigate the beneficial effects of ONS on pancreatic and bile duct cancer, patients receiving chemotherapy, daily intakes of energy, carbohydrates, proteins, and lipids at 8 weeks and after were significantly increased compared to the baseline. Bodyweight, fat-free mass, skeletal muscle mass, body cell mass, and fat mass increased in the ONS group but decreased in the non-ONS group in the first cycle of chemotherapy [95]. If bowel function is normal, but the patient is unable to receive recommended energy orally, total or integral enteral nutrition (EN), should be considered [93,96]. If EN is impossible due to impaired digestive function and symptoms (e.g., diarrhea, nausea, vomiting) or the patient’s lack of consent to EN, parenteral nutrition (PN) is required to provide nutritional support [92,93,97,98]. In order to prevent the occurrence of the reefing syndrome in severely malnourished patients, it is recommended to slowly increase the nutrition energy and monitor the clinical and metabolic stability [98].

### 5.2. Nutrition

Recommended total energy expenditure of cancer patients according to ESPEN guidelines is between 25 and 30 kcal/kg/day with protein intake between 1–1.5 g/kg/day and increased ratio of energy from fat to energy from carbohydrates in patients with insulin resistance. Minerals and vitamins should be administered equally to the RDA. To support muscle mass and metabolism, the maintenance of physical activity with individualized resistance in combination with aerobic exercise is recommended. The supplementation with long-chain N-3 fatty acids or fish oil during chemotherapy in malnourished (or at risk of malnutrition) patients with advanced cancer is suggested to improve appetite and food intake and help in maintaining body weight and lean body mass. Oral or enteral immunonutrition (containing N-3 fatty acids, arginine, and nucleotides) is recommended in upper GI cancer patients as part of perioperative care [98].

## 6. Conclusions

The link between gut microbiota and pancreatic cancer has been intensively analyzed during the last several years. Alterations of gut microbiota affect pancreatic carcinogenesis. Microbes may affect the tumorigenic pathway. The supplementation of gut microbiota with methods, such as administration of prebiotics, probiotics, next-generation probiotics, synbiotics, and fecal microbiota transplantation may open new therapeutic strategies for pancreatic cancer patients. However, there is still a need for new clinically-orientated studies addressing these matters and confirming their efficiency.

## Figures and Tables

**Table 1:** The studies registered in the ClinicalTrials.gov system and regard pancreatic cancer and gut microbiome-associated aspects.

Title of Project	ClinicalTrials.gov	Participants (n)	Conditions	Treatment	Study Type	Locations	Current Status
The Mechanism of Enhancing the Anti-tumor Effects of CAR-T on PC by Gut Microbiota Regulation	NCT04203459	80	Pancreatic cancer	nd	Observational	The first affiliated hospital of Harbin medical university Harbin, Heilongjiang, China	Recruiting
MS-20 on Gut Microbiota and Risk/Severity of Cachexia in Pancreatic Cancer Patients	NCT04600154	40	Pancreatic cancer	Drug: MS-20 Other: Placebo	Interventional	Department of Internal Medicine, National Taiwan University Hospital Taipei, Taiwan	Recruiting
Gut Microbiome Modulation to Enable Efficacy of Checkpoint-based Immunotherapy in Pancreatic Adenocarcinoma	NCT03891979	nd	Pancreatic cancer	Drug: Pembrolizumab Drug: Ciprofloxacin 500 mg PO BID days 1–29 Drug: Metronidazole 500 mg PO TID days 1–29	Interventional	NYU Langone Health New York, NY, USA	Withdrawn
ARGONAUT: Stool and Blood Sample Bank for Cancer Patients	NCT04638751	4000	Non-small Cell Lung Cancer Colorectal Cancer Triple NegTriple-Negativencer Pancreas Cancer	Drug: Immunotherapy Drug: Chemotherapeutic Agent	Observational	Persephone Biosciences, Inc. San Diego, CA, USA	Recruiting
Correlation Between Complications After Pancreaticoduodenectomy and Microbiota (COMPAMIC)	NCT04931069	30	Pancreatic cancer	nd	Interventional	nd	Not yet recruiting
Microbial Diversity of Pancreatic Diseases	NCT03809247	330	Pancreatic cancer Pancreatic diseases	nd	Observational	Ruijin Hospital Shanghai, Shanghai, China	Not yet recruiting
Microbiome Analysis in esoPhageal, PancreatIc and Colorectal Cancer Patients Undergoing Gastrointestinal Surgery (MA-PPING)	NCT04189393	60	Gastrointestinal Cancer Colorectal Cancer Pancreatic Cancer Esophageal Cancer Rectum Neoplasm Esophageal Neoplasms Pancreatic Ductal Adenocarcinoma Colonic Neoplasms	nd	Observational	Nd	Not yet recruiting
The Microbiome of Pancreatic Cancer: “PANDEMIC” Study (PANDEMIC)	NCT04274972	20	Pancreas Cancer Pancreas Infection Pancreas; Fistula	Diagnostic Test: Microbiome evaluation	Observational	AOUI Verona Verona, Italy	Recruiting
Volatiles in Breath and Headspace Analysis—Diagnostic Markers (Volatolome)	NCT03228095	3000	Tuberculosis Gastric Cancer Peptic Ulcer Atrophic Gastritis Intestinal Metaplasia Gastric Dysplasia Colorectal Cancer Colorectal Polyp Colorectal Adenoma Pancreatic Cancer Pancreatitis, Chronic Liver Cancer Liver Cirrhosis Flu, Human Other Infectious Diseases Inflammatory Bowel Diseases	Diagnostic Test: VOC detection in breath and skin headspace Diagnostic Test: Breath sampling Procedure: Upper endoscopy with biopsies Procedure: Colonoscopy with biopsies Procedure: Whole blood/Plasma/serum sampling Diagnostic Test: Faecal sampling Procedure: Histological examination of the surgical specimen Diagnostic Test: Headspace analysis for biological material	Observational	University of Latvia Riga, Latvia	Enrolling by invitation
Nutrition in Gastrointestinal Tumors (NutriGIT)	NCT04476082	80	Pancreatic Cancer Oesophageal Cancer Colon Cancer Liver Cancer Rectal Cancer Bile Duct Cancer GIST, Malignant Neuroendocrine Tumors Small Intestine Cancer Gastric Cancer	nd	Observational	University Medicine Greifswald Greifswald, Germany	Recruiting

nd: “no data”.
